# A Case Report: Ethambutol Causes a Rare Adverse Effect of Peripheral Neuropathy

**DOI:** 10.7759/cureus.23782

**Published:** 2022-04-03

**Authors:** Dhara Rana, Shriya Patel, Trinava Roy, James W Bailey

**Affiliations:** 1 Department of Physical Medicine and Rehabilitation, Rowan University School of Osteopathic Medicine, Stratford, USA; 2 Department of Internal Medicine, Rowan University School of Osteopathic Medicine, Stratford, USA; 3 Department of Physical Medicine and Rehabilitation, Neuromusculoskeletal Institute at Rowan University, Stratford, USA

**Keywords:** lumbosacral radiculopathy, rifampin, clarithromycin, peripheral neuropathy, ethambutol, mycobacterium gordonae

## Abstract

*Mycobacterium gordonae* is a slow-growing acid-fast bacilli mycobacterium with low pathogenic potential. Patients with this infection are treated with antimycobacterial agents such as ethambutol, clarithromycin, and rifampin. We present a rare side effect of ethambutol causing peripheral neuropathy, along with regression of this upon discontinuation of the inciting medication. A 78-year-old male with a past medical history of lumbar degenerative disc disease and lumbosacral radiculopathy presented to the clinic with three weeks of progressively worsening rhinorrhea, nasal congestion, and productive cough with yellow sputum. After a bronchoalveolar lavage (BAL) and a chest computed tomography (CT) scan, he was diagnosed with an *M. gordonae* infection. He was started on a 12-month triple regimen of rifampin, clarithromycin, and high-dose ethambutol. During the first three months of antibiotic therapy, the patient began to have symptoms of gastrointestinal upset and worsening numbness in bilateral lower extremities, especially at night. Because he was unable to tolerate these adverse effects, the patient stopped taking these medications three months into his 12-month course. Upon stopping the antimycobacterial therapy, the patient’s neuropathy began to return to baseline. Based on imaging, electromyography (EMG), nerve conduction studies (NCS), and a literature search of antimycobacterial medicines, we concluded that the high dose of ethambutol is the most likely cause of this patient’s peripheral neuropathy. An important takeaway is that while ethambutol is a well-known cause of optic neuritis, it may also lead to peripheral neuropathy, which may regress upon discontinuation of the medication.

## Introduction

Of all the mycobacteria species, *Mycobacterium gordonae* is a slow-growing acid-fast mycobacterium and is commonly isolated from soil and water [1,2]. Typically, *M. gordonae* is considered non-pathogenic, but its isolation is typically regarded as a contaminant [1-3]. Despite its non-virulent nature, there have been reports of clinically significant diseases caused by *M. gordonae*, especially in patients with underlying predisposition or immunosuppression such as AIDS, steroid therapy, or carcinoma [3]. For the treatment of *M. gordonae*, antimycobacterial agents are most often used, such as ethambutol, clarithromycin, and linezolid [3].

In particular, ethambutol has several well-known adverse effects, including optic neuritis, loss of red/green color discrimination, and loss of visual acuity [3]. Scarce studies in the literature have reported a rare side effect of peripheral neuropathy with ethambutol treatment [4-6]. Peripheral neuropathy is often categorized into large-fiber and small-fiber involvement. In large-fiber peripheral neuropathy, there is loss of joint position, vibration, and sensory ataxia, while in small-fiber peripheral neuropathy there is impairment of pain, temperature, and autonomic functions [7].

We present a unique case of an elderly patient with chronic lumbosacral radiculopathy who developed a large-fiber peripheral neuropathy after starting antibiotic therapy for an *M. gordonae* pulmonary infection. Our patient's symptoms improved after the cessation of medical therapy, including ethambutol.

## Case presentation

**Figure 1:**
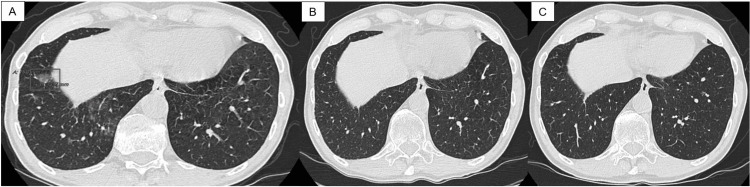
(A) Chest CT in January 2020 before the triple antibiotic therapy for the mycobacterial lung infection initiated; 15 mm × 12 mm ground glass nodule is outlined in the right lower lobe; (B) chest CT in August 2020 after three months of triple antibiotic therapy; (C) chest CT in February 2021 after nine months of discontinuing the triple antibiotic therapy CT: computerized tomography

Our patient is a 78-year-old male with a past medical history of prostate adenocarcinoma status post-radiation therapy, lumbar degenerative disc disease, lumbosacral radiculopathy, cervical spondylosis, and right ankle and foot osteoarthritis who presented to the office with three weeks of rhinorrhea, nasal congestion, and cough with yellow productive sputum. He stated that these symptoms had progressively worsened in the last three weeks. The patient’s physical examination revealed wheezing in the left and right lower lung fields. These findings raise questions about the acute bronchitis of an unspecified organism. The patient underwent a chest CT without contrast, which revealed a 15 mm × 12 mm ground glass nodule in the right lower lobe (Figure 1A) and ground-glass opacities in both lower lobes. Endobronchial fine-needle aspiration of the lung lymph nodes was negative for malignant cells. Later, the patient underwent bronchoalveolar lavage (BAL) of the right lower lung lobe. The culture and stain of the BAL revealed acid-fast bacilli. The DNA probe result of BAL from the right lower lung lobe revealed a positive for *M. gordonae* and a negative for *M. tuberculosis* complex and *M. avium* complex. He was then started on a 12-month regimen of rifampin 300 mg three times a week, clarithromycin 500 mg BID, and ethambutol 400 mg TID in June 2020. 

The patient followed up in the clinic in September 2020, three months after starting triple antibiotic therapy. He subjectively reported new symptoms of gastrointestinal upset and worsening numbness in his bilateral feet, especially at night. A physical exam at this time demonstrated 5/5 motor strength in bilateral lower extremities from L2-S1, a negative Babinski sign, and a negative ankle clonus. There were absent Achilles deep tendon reflexes (DTR) bilaterally and +2/4 patellar reflexes bilaterally. Sensation to a pinprick was diminished at S1 bilaterally, but proprioception was intact. An x-ray of the lumbar spine in September 2020 demonstrated mild degenerative joint disease and facet arthropathy with no evidence of intraosseous abnormalities. An electromyography (EMG) and nerve conduction studies (NCS) of bilateral lower extremities were subsequently performed. The results were consistent with moderately severe large-fiber peripheral sensory-motor neuropathy with axonal loss. Importantly, this corresponded to the time period in which he started the antibiotic regimen. The EMG also confirmed underlying chronic bilateral L5 and S1 radiculopathy. NCS of the bilateral lower extremities demonstrated reduced amplitude responses in the peroneal and tibial motor nerves, slowed conduction velocity of the right peroneal motor nerve, and an absence of response from the right and left sural sensory nerves. The patient underwent a follow-up chest CT scan in August 2020, which revealed resolution of all previously found ground-glass opacities, including the 15 mm × 12 mm nodule (Figure 1B). Because of this finding and the patient's worsening neuropathy, the infectious disease physician agreed to stop any further antibiotic treatment.

Seven months after stopping treatment, the patient was seen in the physical medicine and rehabilitation (PM&R) clinic in April 2021. At this visit, he reported that his lower extremity numbness had returned to his baseline level. On a physical exam, sensation to light touch was intact in both lower extremities, but there was decreased sensation to pinprick in the S1 distribution. DTR were +2/4 in both lower extremities except for +1/4 at the right Achilles. There was 5/5 bilateral lower extremity motor strength from L2-S1, negative Hoffman’s sign, and negative ankle clonus. For continued symptomatic relief, he was started on gabapentin 100 mg 1 tab/day. Three months later, the patient followed up in the clinic, and he noted an improvement in his symptoms after taking gabapentin as prescribed. Thus, after nine months without triple therapy, the patient subjectively noticed an improvement in his neuropathic symptoms.

Radiology findings

The patient underwent a chest CT in January 2020, and it showed a 15 mm × 12 mm ground glass nodule in the right lower lung lobe and ground-glass opacities in both lungs, suggesting either an infectious or inflammatory etiology (Figure 1A). After three months of antibiotic therapy, the patient underwent a follow-up chest CT in August 2020, which demonstrated no suspicious pulmonary nodules, and the previous 15 mm × 12 mm ground glass nodule in the right lower lobe was resolved (Figure 1B). Without undergoing an additional nine months of antibiotic therapy, the patient had another follow-up chest CT in February 2021, which showed no masses, consolidations, or suspicious nodules, but did show residual bi-apical pleural-parenchymal scarring (Figure 1C).

## Discussion

*M. gordonae*, formerly called *Mycobacterium aquae*, was first described in 1962 as a slow-growing acid-fast bacilli mycobacterium that can be found in freshwater, pipelines, and laboratory faucets [1-3]. A recent large epidemiological study based in a clinically isolated area in over 60 laboratories showed that *M. gordonae* was the second most frequently identified species [2]. Despite its ubiquitous nature, *M. gordonae* is nonpathogenic [8]. *M. gordonae* does not grow in ordinary culture media and its development is detected only after nine to twelve days [1]. Some of the common symptoms of *M. gordonae* pulmonary infection include cough, weight loss, dyspnea, hemoptysis, and fever [8,9]. The presence of clinical symptoms, pulmonary physical exam, and radiographic abnormalities are essential for the diagnosis of *M. gordonae* pulmonary infection.

To make a diagnosis of non-tuberculous mycobacteria (NTM) pulmonary disease, one must ensure all clinical and radiologic criteria are fulfilled and at least one microbiological criterion is as well. The clinical and radiologic criteria that must be met are (1) pulmonary or systemic symptoms, (2) nodular or cavitary opacities on chest radiographs, or bronchiectasis with multiple small nodules on high-resolution computed tomography, and (3) appropriate exclusion of other diagnoses [3]. In addition, at least one of the following microbiologic criteria must be fulfilled: (1) positive culture from at least two separate expectorated sputum samples or (2) positive culture results from at least one bronchial wash or lavage or (3) transbronchial or another lung biopsy with mycobacterial histologic feature and positive culture for NTM; or biopsy showing mycobacterial histopathologic features and one or more sputum or bronchial washings that are culture positive for NTM [3]. Our patient met all the clinical and radiological criteria and had a positive result from the bronchial wash which revealed *M. gordonae* via DNA probe.

A variety of radiographic findings are possible, including consolidation, pulmonary nodules, and cavities. A retrospective study describing CT findings of NTM pulmonary infection showed bilateral small nodules, cylindrical bronchiectasis, and branching centrilobular nodules regardless of the specific infective mycobacterial species [10]. In addition to the clinical symptoms and radiological findings, isolation of NTM in culture from sputum is essential for the diagnosis of NTM pulmonary infection. However, in the absence of clinical and radiographic evidence, positive cultures from the sputum and bronchial wash should be interpreted with caution because these cultures can be contaminated [9].

The treatment regimen for *M. gordonae* infection is not well defined, but antimicrobial agents that are most consistently active in vitro include ethambutol, rifabutin, clarithromycin, linezolid, and fluoroquinolones [3,11]. *M. gordonae* is resistant to isoniazid [8,12]. The duration of therapy is also ill-defined, and in one review, the therapy ranged from nine to twenty-two months with a median of fifteen months [8,12]. Our patient has been prescribed a 12-month regimen of three antibiotic therapies, including ethambutol (400 mg TID), clarithromycin (500 mg BID), and rifampin (300 mg three times a week). However, because he developed worsening symptoms of peripheral neuropathy on top of his baseline chronic lumbosacral radiculopathy, he discontinued the antibiotic regimen after three months and refused to continue for an additional nine months. The patient’s EMG confirmed that this peripheral neuropathy coincided with the initiation of triple antibiotic therapy. Interestingly, a repeat CT scan showed an improvement in the mycobacterium nodule, and the patient’s infectious disease physician concluded that he did not need to complete an additional nine months of therapy (Figure 1B). With the cessation of therapy, the patient’s lower extremity neuropathy returned to his baseline level. In addition, a thoracic spine MRI in January 2020 demonstrated no evidence of new malignancy or metastasis, and a lumbosacral spine X-ray in September 2020 showed only mild degenerative disease without intraosseous abnormalities.

Because his exacerbated symptoms occurred after taking ethambutol, we proposed that the two were related. To confirm that ethambutol caused the adverse effect that was seen on the EMG and NCS, we used the Naranjo adverse drug reaction (ADR) probability scale to rule out other causes of his new-onset peripheral neuropathy. The Naranjo ADR scale was developed to help standardize the assessment of the causality of adverse drug reactions and to ensure that a complication did not manifest from a disease [13,14]. In our case, the Naranjo ADR score was 7 out of 13, indicating a probable ADR. This indicates that the "reaction followed a reasonable temporal sequence after a drug, followed a recognized response to the suspected drug, was confirmed by withdrawal but not by exposure to the drug, and could not be reasonably explained by the known characteristic of the patient’s clinical state" [15]. We answered "yes" to questions 1, 2, 3, and 10, "no" to question 5, and "not known or not done" to questions 4, 6, 7, 8, and 9.

Despite the regression of his peripheral neuropathy, the question begets what medication from the antibiotic regimen caused it. One likely culprit for this is ethambutol. While there is extensive literature evidence that shows ethambutol’s association with optic neuritis, there are several studies that demonstrate its association with reversible, distal sensory polyneuropathy [16,17]. Although the mechanism of ethambutol causing reversible sensory neuropathy is unclear, an animal study showed high levels of ethambutol cause severe neurotoxic effects resulting in unsteady gait, loss of equilibrium, and other disturbances in coordination [4]. Furthermore, the pathogenesis of ethambutol causing reversible neuropathy can be due to similar mechanisms seen in optic neuritis from ethambutol. The pathogenesis of ethambutol-induced optic neuritis is from zinc binding to ethambutol’s -NH groups. This decreases zinc availability for its vital function as a cofactor for various important enzymes, such as lactic acid dehydrogenase and others involved in neural metabolism [4].

Although there are a limited number of studies, cases have been reported in the literature of peripheral neuropathy associated with ethambutol [4-6]. These studies all revealed that patients developed peripheral neuropathy while taking ethambutol. This was evident in NCS, which showed diminished sensory conduction, motor conduction, or both [5], in addition to absent nerve conduction [6]. One study showed a gradual improvement in NCS findings over the course of 26 months after stopping supratherapeutic doses of ethambutol [5]. This study showed that a patient with no pre-existing neuropathy developed distal sensory polyneuropathy after mistakenly being prescribed five times the usual maximum dose. A physical exam showed hyperactive DTR, ankle clonus, and Hoffman’s sign, which subsequently resolved after discontinuing the ethambutol [5]. Similarly, our patient subjectively noticed that after stopping his antibiotic regimen, he had milder symptoms of peripheral neuropathy in addition to his baseline lumbosacral radiculopathy symptoms.

A randomized study investigated lower extremity numbness in 7 out of 187 patients who received ethambutol. The authors found a higher incidence of paresthesias among patients who received ethambutol either as a fixed dose of 1 g daily or 25 mg/kg of body weight per day [18]. Furthermore, other case reports published in the literature showed an adverse effect of peripheral neuropathy in patients who received 13-50 mg/kg of ethambutol daily [4,6]. Similarly, our patients received 1200 mg per day of ethambutol (400 mg TID), which is supported by the latter study. Although optimal dosing for NTM other than *Mycobacterium avium* complex has not been determined, the general recommended dosage for *M. kansasii*, which is also an NTM, is 15 mg/kg/day, which equals to 1050 mg daily for our 70 kg patient [3].

There are scarce studies demonstrating rifampin and clarithromycin causing peripheral neuropathy, and thus these medications are unlikely culprits in this patient’s peripheral neuropathy. Furthermore, the general recommended dosage for clarithromycin is 500 mg BID, and for rifampin, it is 600 mg three times weekly as part of an appropriate combination regimen. Our patient was given an appropriate dosage of clarithromycin (500 mg BID) and an underdose of rifampin (300 mg three times a week), making these two medications unlikely to have led to his neuropathy. The only NTM medication that our patient was given a high dose of was ethambutol, 1200 mg daily.

Ultimately, of the three antimycobacterial medications, ethambutol may be the reason behind this patient’s peripheral neuropathy. An important take-away from this patient’s presentation is to consider other side effects of ethambutol, specifically its role in causing peripheral neuropathy. It is well known that ethambutol is a common culprit behind optic neuritis, but it is necessary to consider other adverse effects of this medication, like peripheral neuropathy. In addition, it is crucial to adhere to appropriate dosing regimens of ethambutol because overtreatment can lead to a new onset of peripheral neuropathy, potentially highlighting a dose-dependent effect. Physicians should keep this in mind when prescribing ethambutol to patients who already have some kind of neuropathy, like radiculopathy in our patients. In prior cases reported in the literature, ethambutol-induced peripheral neuropathy was reversed upon cessation of ethambutol [4-6], and this was seen in our patient as well. Thus, the most important point our case demonstrates is that peripheral neuropathy may regress upon complete discontinuation of ethambutol, similar to the findings in the literature. 

In this case, the management of new-onset peripheral neuropathy from ethambutol is to first stop the medication to halt any further damage. In addition to the cessation of medication, vitamin B6 supplementation can also help with generalized peripheral neuropathy [19]. Also, there is the possibility of co-prescribing pyridoxine (vitamin B6 supplement) along with ethambutol. While this is already commonplace with isoniazid for preventing peripheral neuropathy, it may be worthwhile for further research studies to focus on the relationship between ethambutol and pyridoxine. Our patient subjectively noted significantly reduced neuropathy after cessation of the medication, and the introduction of gabapentin further helped manage his existing symptoms.

## Conclusions

Our case report demonstrates that ethambutol is a potential culprit for a rare adverse effect of peripheral neuropathy. An important takeaway from our paper highlights is that once this drug was discontinued, the patient's peripheral neuropathy regressed, and his symptoms returned to their baseline. Our patient had also been prescribed a high dose of ethambutol, which contributed to this presentation, demonstrating that this may be a dose-dependent effect. While this rare side effect of ethambutol has been reported in the literature, it is otherwise not widely known to medical providers. The conclusion of this paper, therefore, adds a rare and otherwise not well-known side effect of ethambutol to the medical literature. This adverse effect should be taken into consideration before prescribing ethambutol therapy to patients with pre-existing neuropathy. 
